# Complete Genome Sequence of a Bluetongue Virus Serotype 15 Strain Isolated from China in 1996

**DOI:** 10.1128/genomeA.00557-18

**Published:** 2018-06-28

**Authors:** Defang Liao, Heng Yang, Lei Xiao, Jianbo Zhu, Jinpin Wang, Zhanhong Li, Nianzu Zhang, Huachun Li

**Affiliations:** a Yunnan Tropical and Subtropical Animal Viral Disease Laboratory, Yunnan Animal Science and Veterinary Institute, Yunnan, People’s Republic of China

## Abstract

The full-genome sequence of bluetongue virus serotype 15 (BTV-15) strain B105/YN/1996 isolated in China was determined for the first time. The virus was isolated from sentinel cattle in Yunnan Province, China, in 1996. The total size of the BTV-15 strain B105/YN/1996 genome is 19,161 bp in length. Phylogenetic analyses demonstrate that it belongs to the major eastern BTV topotype. This work is the first to document the complete genomic sequence of a BTV-15 strain from China. The sequence information will help determine the geographic origin of Chinese BTV-15 and provide data to facilitate future analyses of the genetic diversity and phylogenetic relationships of BTV strains.

## Abstract (precis)

The full-genome sequence of bluetongue virus serotype 15 (BTV-15) strain B105/YN/1996 isolated in China was determined for the first time. The virus was isolated from sentinel cattle in Yunnan Province, China, in 1996.

## GENOME ANNOUNCEMENT

Bluetongue virus (BTV) is the type species of the genus Orbivirus, within the family Reoviridae (1). The genome of BTV consists of 10 linear double-stranded RNA segments encoding seven structural proteins (VP1 to VP7) and four nonstructural proteins (NS1, NS2, NS3/NS3a, and NS4) (2, 3). To date, 27 BTV serotypes have been described worldwide (4–6). In China, BTV was first isolated from sheep with severe BT clinical signs during an outbreak in Shizhong County, Yunnan Province, China, in 1979, and this strain was later identified as serotype 1 (7). During the Chinese BTV epidemiological investigations conducted during the years 1996 and 1997, 108 BTV strains belonging to eight serotypes (BTV-1, -2, -3, -4, -9, -12, -15, and -16) were isolated from the blood of sentinel cattle (8, 9).

Geographical separation over long periods of time has allowed bluetongue viruses in different regions to acquire unique point mutations, some of which may make them particularly well suited to transmission and survival in their local ecosystems (10). BTV strains show nucleotide sequence variations that reflect their origins from different geographic regions around the world, with a clear division of most genome segments between eastern and western topotypes (11, 12). Although full-genome sequences are available for four BTV-15 isolates in GenBank, two of them from South Africa and another two from Australia (13, 14), there have been no reports describing the full genomic sequences of BTV-15 strains isolated from China or other locations. It is necessary to acquire and analyze additional complete genomic sequences of BTV-15 strains to study the molecular features of BTV-4 strains and establish phylogenic relationships among BTV serotypes.

We report here the complete genomic sequence of the BTV-15 strain B105/YN/1996, isolated from blood of sentinel cattle in Mangshi, Dehong District of Yunnan Province, China, in 1996. Viral double-stranded RNA preparation, full-length cDNA synthesis, PCR amplification, and sequencing of B105/YN/1996 were performed according to the protocol described by Yang et al. (15).

The sizes (in base pairs) of B105/YN/1996 segments 1 to 10 are 3,944, 2,909, 2,772, 1,981, 1,763, 1,639, 1,154, 1,125, 1,052, and 822, respectively. They encode proteins with amino acid lengths as follows: VP1, 1,302; VP2, 952; VP3, 901; VP4, 644; VP5, 552; VP6, 330; VP7, 349; NS1, 527; NS2, 354; and NS3/NS3a, 229/216. Further phylogenetic analyses of the sequence show that B105/YN/1996 is a grouping within the major eastern BTV topotype. The data presented here are the first to be reported for the complete sequence of a native BTV-15 isolated in China that belongs to the eastern strain. This information will facilitate future investigations of the molecular characteristics and geographic origins of BTV-15 strains from China and from other countries.

### Accession number(s).

The full genomic sequence of the BTV-15 strain B105/YN/1996 was deposited in GenBank. The accession numbers MH346491 to MH346500 correspond to B105/YN/1996 segments 1 through 10.
